# The role of SARS-CoV-2 nucleocapsid protein in antiviral immunity and vaccine development

**DOI:** 10.1080/22221751.2022.2164219

**Published:** 2023-03-01

**Authors:** Haiyun Yu, Fei Guan, Heather Miller, Jiahui Lei, Chaohong Liu

**Affiliations:** a Department of Pathogen Biology, School of Basic Medicine, Tongji Medical College, Huazhong University of Science and Technology, Wuhan, People’s Republic of China; b Laboratory of Intracellular Parasites, Rocky Mountain Laboratories, National Institute of Allergy and Infectious Diseases, National Institutes of Health, Hamilton, MT, USA

**Keywords:** SARS-CoV-2, nucleocapsid, inflammation, cell death, innate immunity, adapted immunity, vaccine

## Abstract

The coronavirus disease 2019 (COVID-19) has caused enormous health risks and global economic disruption. This disease is caused by the severe acute respiratory syndrome coronavirus 2 (SARS-CoV-2). The SARS-CoV-2 nucleocapsid protein is a structural protein involved in viral replication and assembly. There is accumulating evidence indicating that the nucleocapsid protein is multi-functional, playing a key role in the pathogenesis of COVID-19 and antiviral immunity against SARS-CoV-2. Here, we summarize its potential application in the prevention of COVID-19, which is based on its role in inflammation, cell death, antiviral innate immunity, and antiviral adaptive immunity.

## Introduction

The coronavirus disease 2019 (COVID-19) has caused global economic disruption and enormous health risks. According to the information provided by the World Health Organization, by 15th September 2022, there were more than 607 million confirmed cases and over 6.49 million deaths worldwide [1]. This disease is caused by the severe acute respiratory syndrome coronavirus 2 (SARS-CoV-2). The typical clinical symptoms of COVID-19 include fever, dry cough, dyspnea, muscle pain, fatigue, and even death. Besides the respiratory systems, COVID-19 may affect multiple organs and tissues. Intriguingly, the involvement of other organs and tissues may be independent of the severity of the respiratory disease [2].

SARS-CoV-2, which is an enveloped, positive and single-stranded RNA virus, belongs to the β-Coronavirus family, which is in the same family as the middle east respiratory syndrome coronavirus (MERS-CoV) and SARS-CoV. The other human-infecting coronaviruses (HCoV) are the low-pathogenicity members HCoV-OC43, HCoV-NL63, HCoV-HKU1, and HCoV-229E. Many host proteins are essential for viral entry. Among them is the transmembrane serine protease 2 (TMPRSS2), which cleaves and thereby activates the SARS-CoV-2 surface glycoprotein, spike (S) protein. Activated S protein recognizes and then binds to angiotensin-converting enzyme 2, which is the SARS-CoV-2 receptor on the host cell. The binding of the S protein to angiotensin-converting enzyme 2 induces the fusion of the virus with the host cell membrane [3]. After SARS-CoV-2 enters the host cell, RNA-dependent RNA polymerase replicates and transcribes the ∼30kb viral genome, leading to the production of 4 structural proteins, i.e., envelope (E), membrane (M), nucleocapsid (N), and S proteins, 9 putative accessory proteins, and the 16 non-structural proteins (Nsp1-Nsp16) [4].

The main effect of the N-protein is to integrate the viral genomic RNA into a ribonucleoprotein complex, promoting the M and E proteins to initiate viral assembly [4]. There are enormous evidences indicating that the N-protein is multi-functional with many roles. Here, we focus on its function in inflammation, antiviral innate immunity, antiviral adaptive immunity and cell death.

## RNA-induced liquid–liquid phase separation (LLPS) of SARS-CoV-2 N-protein

**Figure 1.**
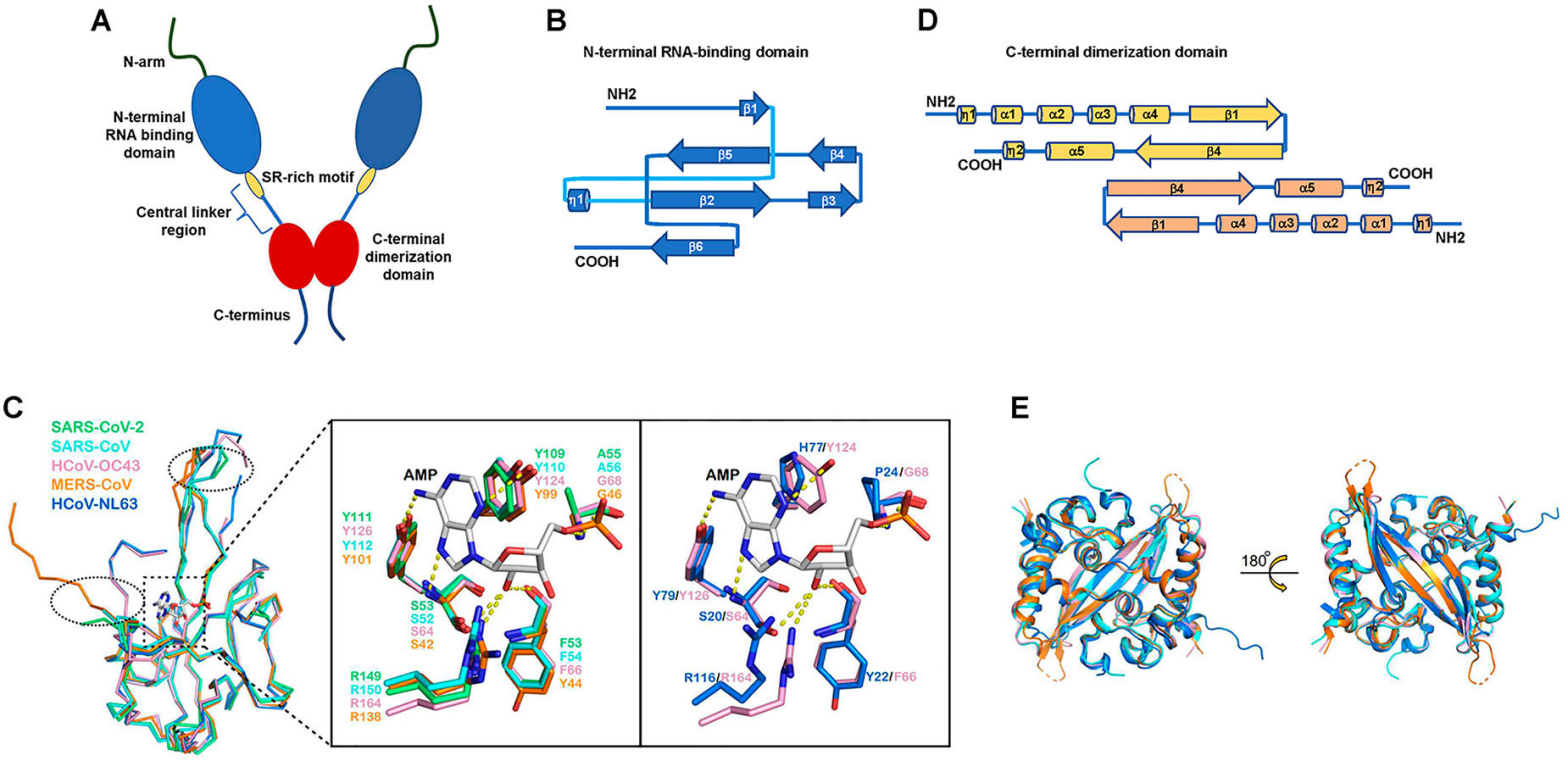
The structure of SARS-CoV-2 N-protein. (A). Model of SARS-CoV-2 N-protein dimer. (B). Topology diagram for SARS-CoV-2 N-protein N-terminal RNA-binding domain. (C). Superimposed structures in ribbon representation of N-protein N-terminal RNA-binding domain from SARS-CoV-2 (green), SARS-CoV (cyan), HCoV-OC43 (pink), MERS-CoV (orange), and HCoV-NL63 (blue) [6]. The loops that show significant differences are highlighted by dotted circles. The AMP ligand in the HCoV-OC43 N-protein N-terminal RNA-binding domain-AMP complex is shown as a stick structure. D. Topology diagram for SARS-CoV-2 N-protein C-terminal dimerization domain. E. Superimposed structures in ribbon representation of N-protein C-terminal dimerization domain from SARS-CoV-2 (pink), SARS-CoV (cyan), MERS-CoV (orange) and HCoV-NL63 (blue). Figure adapted from [6].

Coronavirus N-proteins have a C-terminal dimerization domain and a N-terminal RNA-binding domain. The two structural domains are connected by an intrinsically disordered central linker region with a Ser/Arg (SR)-rich motif that contains putative phosphorylation sites [5–7]. Moreover, the N-terminal domain (NTD) and C-terminal domain (CTD) are flanked by intrinsically disordered N-arm and C-terminus, respectively (Figure 1(A)) [5,6]. NTD of SARS-CoV-2 N-protein has a right-handed fist shape with a four-stranded antiparallel β-sheet core subdomain, sitting between short 3_10_ helices (loops) and a protruding β-hairpin region formed by β3 and β4 strands out of the core (Figure 1(B)) [5,6]. Comparison of its structure with other human-infecting coronaviruses N-protein NTD structures indicates that the orientations of the N-terminal loops, the top tip of the protruding region, the bottom of the core subdomain are distinct (Figure 1(C)) [5,6]. SARS-CoV-2 N-protein CTD forms a tight homodimer with an overall rectangular slab shape. Each contains five α-helices, two antiparallel β-strands, and two 3_10_ helices (Figure 1(D)) [6,7]. The overall structure of SARS-CoV-2 N-protein CTD is highly similar to the available N-protein CTD structures of SARS-CoV, MERS-CoV and HCoV-NL63 (Figure 1(E)). Interestingly, the electrostatic potential surfaces at the helical side of the dimer show distinct features [6,7], suggesting novel roles of SARS-CoV-2 N-protein CTD in viral RNA binding and transcriptional regulation. The unique structural characteristics of SARS-CoV-2 N-protein might contribute to the unique roles of SARS-CoV-2 N-protein in COVID-19 pathogenesis, as discussed below.

The SARS-CoV-2 N-protein has a net positive charge, which allows it to bind the negatively charged viral genome RNA and assemble it into a higher ordered structure. After binding to RNA, the SARS-CoV-2 N-protein robustly goes through a liquid–liquid phase separation, forming liquid-like droplets or condensates. LLPS of the SARS-CoV-2 N-protein depends on the RNA sequence and structure [8]. This process is enhanced by body temperature and modulated by ionic strength and RNA concentration [8,9]. The intrinsically disordered N-arm, the central linker region, and the C-terminal dimerization domain are essential for robust LLPS [9].

When the cell is under stress, stress granules are formed in the cytoplasm through LLPS, which contain translationally stalled mRNAs. SARS-CoV-2 N-protein can be recruited into phase-separated forms of human stress granule-associated heterogeneous nuclear ribonucleoproteins (hnRNPs) [9–12]. Specifically, the SARS-CoV-2 N-protein targets a conserved surface groove of the nuclear transport factor 2-like domain of Ras GTPase-activating protein-binding protein 1 (G3BP1), a stress granule assembly factor [10]. Through its N-arm, the SARS-CoV-2 N-protein partitions into stress granules, modulates the disassembly of stress granules, stimulates the aggregation of stress granule-related amyloid protein, and promotes amyotrophic lateral sclerosis-associated amyloid aggregation [9–12].

## Induced inflammation by SARS-CoV-2 N-protein

Intracellular SARS-CoV-2 N-protein has a key role in the production of proinflammatory cytokines. SARS-CoV-2 infection leads to inflammasome activation and intense inflammasome formation is associated with fatal COVID-19 [13,14]. SARS-CoV-2 N-protein directly binds to sensor protein NOD-like receptor family PYRIN domain containing-3 (NLRP3) and promotes the interaction between NLRP3 and adaptor protein apoptosis-associated speck-like protein containing a caspase recruitment domain (ASC). This facilitates ASC oligomer formation, which provides a platform for Caspase1 activation [15]. Activated Caspase1 cleaves pro-IL-1β and Gasdermin family member D (GSDMD). Cleaved GSDMD oligomerizes to form pores, causing cell death termed pyroptosis with cell membrane permeability and cell content leakage [13–15]. The augmented assembly of NLRP3 inflammasome upon SARS-CoV-2 N-protein expression results in cytokine production and lung injury in mice [15]. Additionally, SARS-CoV-2 infection induces the enhancement of nuclear factor-κB (NF-κB) and mitogen-activated protein kinase (MAPK) signaling pathways. SARS-CoV-2 N-protein expression enhances NF-κB activation in Calu-3 human lung cancer cells, HEK-293 human embryonic kidney epithelial cells, and Huh7 human hepatocellular carcinoma cells and its role is blocked by LLPS inhibitor 1,6-hexanediol. Together with viral RNA, the NF-κB pathway upstream kinases, IKK complex and TAK1, are recruited to N-protein condensates *in vitro* [16]. Therefore, LLPS of SARS-CoV-2 N-protein provides a platform for NF-κB hyperactivation. Furthermore, SARS-CoV-2 N-protein directly binds to the Smad3 protein in respiratory epithelial cells [17], similar to SARS-CoV N-protein [18]. The complex of SARS-CoV-2 N-protein-Smad3 could enter the nuclei and augment miR-145 expression, which down-regulates the expression of cystic fibrosis transmembrane conductance regulator (CFTR), a cAMP-dependent anion channel responsible for transepithelial Cl^−^ transport [17]. In respiratory epithelial cells, down-regulation of CFTR induces activation of serum glucocorticoid regulated kinase 1 (SGK1), a Cl^−^-sensing protein that promotes airway inflammation [17]. Moreover, SARS-CoV-2 N-protein augments phosphodiesterase 4 (PDE4) expression, which leads to cAMP degradation and sustained CFTR dysfunction. These mechanisms aggravate acute lung injury. The NF-κB inhibitor, PDTC, reverses PDE4 up-regulation and intracellular cAMP reduction. Also, the selective PDE4 inhibitor, Rolipram, counters airway inflammation by reducing the level of intracellular Cl^−^ [17].

Intriguingly, the SARS-COV-2 N-protein is found in the serum within two weeks of the post onset of symptoms or diagnostic PCR. Serum N-protein level is correlated with a COVID-19 patient's inflammatory response, tissue damage, coagulation, and disease severity [19–21]. Extracellular SARS-CoV-2 N-protein also contributes to inflammation by regulating complement and it has been described that COVID-19 patients show complement hyperactivation [22,23]. The N-proteins from SARS-CoV, MERS-CoV, and SARS-CoV-2 recruit and activate mannose-binding protein-associated serine protease 2 (MASP-2), a key factor in the lectin pathway of complement activation. Blocking the interaction between N-protein and MASP-2 or depletion of MASP-2 can alleviate lung injury *in vivo* and *in vitro* and N-protein-induced complement hyperactivation [22,23]. Furthermore, when the SARS-CoV-2 N-protein is added to the culture medium of human primary lung microvascular endothelial cells, it significantly induces the expression of intracellular adhesion molecule 1 and vascular cell adhesion protein 1, which are markers of endothelial cell activation [24]. Simultaneously, NF-κB and MAPK pathways are activated [24]. Even though N-proteins from different coronaviruses are highly conserved in protein sequences, N-proteins from HCoV-HKU1, SARS-CoV, and MERS-CoV have no such role in endothelial cells [24]. The plasma membrane-bound pattern recognition receptor Toll-like receptor 2 antagonist, CU-CPT22, blocked SARS-CoV-2 N-protein-induced endothelial cell activation [24]. Additionally, incubation of macrophages with SARS-CoV-2 N-protein induces M1 polarization and the production of proinflammatory cytokines [25,26]. Also, incubation of human arterial fibroblasts with SARS-CoV-2 N-protein leads to altered expression of the receptor for the globular head of C1q, which links N-protein-induced inflammation with thrombosis in the vascular system [27]. SARS-CoV-2 N-protein also up-regulates the expression of tissue factor and intracellular adhesion molecule 1, markers involved in vascular coagulation and inflammation, and glucose transporter 4, a diabetic marker [27]. Lastly, complement activation and the activation of signaling pathways through Toll-like receptor 2 might be involved in increasing levels of IL-6 in the lungs of mice inoculated nasally with SARS-CoV-2 N-protein and inducing acute lung injury in mice administrated intratracheally with SARS-CoV-2 N-protein [26,28]. Indeed, the lung injury is associated with NF-κB p65 phosphorylation. Accordingly, an NF-κB inhibitor pyrrolidine dithiocarbamate alleviates SARS-CoV-2 N-protein-induced lung injury [25].

**Figure 2.**
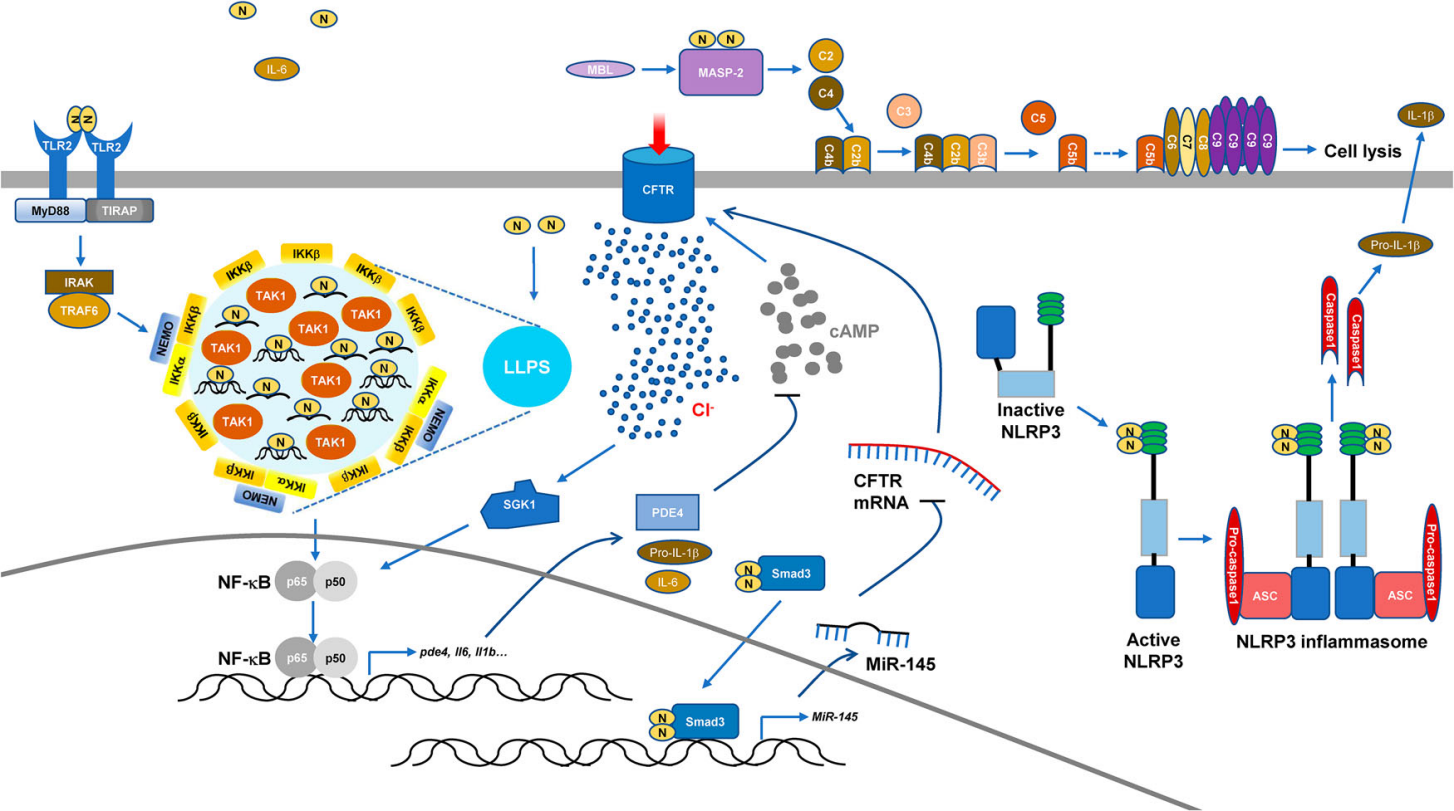
Induced inflammation by SARS-CoV-2 N-protein.

Together, both intracellular and extracellular SARS-CoV-2 N-protein increases inflammation via various mechanisms (Table 1) (Figure 2), which contributes to COVID-19 pathogenesis.

**Table 1.** Effects of different human-infecting coronavirus N-proteins on host cell signaling.

Human-infecting coronavirus	Intracellular N-protein											Extracellular N-protein	
	Interaction w/ NLRP3	Activation of NF-κB	Interaction w/ Smad3	Interaction w/ GSDMD	Interaction w/ PDK1	α-Synuclein aggregation	Interaction w/ Rig-I	Interaction w/ G3BP1	Interaction w/ MAVS	Interaction w/ STAT1/2	Inhibition of RNA interference	Activation of complement	Activation of NF-κB and MAPK
HCoV-OC43	N/A	N/A	N/A	N/A	N/A	N/A	N/A	N/A	N/A	N/A	N/A	N/A	N/A
HCoV-NL63	N/A	N/A	N/A	N/A	N/A	N/A	N/A	N/A	N/A	N/A	N/A	N/A	N/A
HCoV-HKU1	N/A	N/A	N/A	N/A	N/A	N/A	N/A	N/A	N/A	N/A	N/A	N/A	× [24]
HCoV-229E	N/A	N/A	N/A	N/A	N/A	N/A	N/A	N/A	N/A	N/A	N/A	× [22]	N/A
MERS-CoV	N/A	N/A	N/A	N/A	N/A	N/A	√ [29–31]	× [32]	N/A	N/A	√ [33]	√ [22]	× [24]
SARS-CoV	N/A	√ [17]	√ [18]	N/A	N/A	N/A	√ [29–31]	√ [32]	N/A	× [34]	√ [33]	√ [22]	× [24]
SARS-CoV-2	√ [15]	√ [16]	√ [17,35]	√ [36]	√ [37]	√ [38]	√ [29–31]	√ [32,39]	√ [40]	√ [30,41]	√ [42,43]	√ [22,23]	√ [24,25]

Note: w/, with; N/A, non-available.

## Modulation of cell death by SARS-CoV-2 N-protein

Despite that SARS-CoV-2 infection promoting NLRP3 inflammasome activation, IL-1β secretion and cell death is only barely detected during early post-infection. This is due to the SARS-CoV-2 N-protein inhibiting IL-1β secretion and pyroptosis while promoting the cleavage of pro-IL-1β [36]. Yeast two-hybrid screening of SARS-CoV-2 N-protein-interaction candidates identified GSDMD, which is the substrate of Caspase1 and is involved in IL-1β secretion and pyroptosis. The binding of SARS-CoV-2 N-protein to the GSDMD linker prevents GSDMD from being cut by active Caspase1 [36]. Interestingly, SARS-CoV-2 N-protein binds to NLRP3 and promotes Caspase1 activation, which can cleave GSDMD [15], on the other hand N-protein can bind to GSDMD to stop pyroptosis on the same pathway [36]. GSDMD-mediated pyroptosis restricts viral production. The binding of SARS-CoV-2 N-protein to GSDMD ensures enough time for SARS-CoV-2 coronaviruses to reproduce and spread before the immune system attacks, which might explain the long asymptomatic infection of COVID-19. However, SARS-CoV-2 coronaviruses will enter into the lytic phase eventually. Due to SARS-CoV-2 N-protein-NLRP3 interaction, the massive storage of mature IL-1β in the cytosol will lead to the release of abundant inflammatory cytokines, which cause tissue injury. Therefore, SARS-CoV-2 N-protein-NLRP3 interaction and SARS-CoV-2 N-protein-GSDMD interaction act synergistically to contribute to COVID-19 pathogenesis.

The role of SARS-CoV-2 N-protein in apoptosis has also been identified [37]. SARS-CoV-2 M-protein directly induces apoptosis in Vero E6 monkey kidney epithelial cells and HepG2 human hepatocellular carcinoma cells [37]. Mechanistically, M-protein binds to 3-phosphoinositide-dependent protein kinase-1 (PDK1) and thereby hinders the interaction between PDK1 and its substrate protein kinase B (PKB, also named as Akt) [37]. The SARS-CoV-2 N-protein does not directly induce apoptosis in Vero E6 or HepG2 cells [37]. Despite this, SARS-CoV-2 N-protein enhances M-protein-induced apoptosis via binding to both M-protein and PDK1 and strengthening M-protein-mediated attenuation of the PDK1-PKB/Akt interaction [37].

SARS-CoV-2 N-protein is also implicated in uncharacterized types of cell death [35,38]. SARS-CoV-2 can infect the neuron and it has been discovered that *in vitro*, the SARS-CoV-2 N-protein significantly speeds up the aggregation of α-synuclein protein, a key feature of Parkinson's disease [38]. Microinjection of SARS-CoV-2 N-protein into a neuronal cell model (SH-SY5Y cells) leads to less vesicle-bound α-synuclein protein and more cell death [38]. Furthermore, decreased patients who suffered acute kidney injury from SARS-CoV-2 infection had readily detectable N-protein in their renal tubular epithelial cells [35]. Mechanistically, SARS-CoV-2 N-protein interacts with Smad3 and enhances TGF-β1-induced Smad3 phosphorylation [17,35]. Enhanced Smad3 activation leads to p21 expression and cell cycle arrest at the G0/G1-phase, which eventually causes tubular epithelial cell death and acute kidney injury [35].

**Figure 3.**
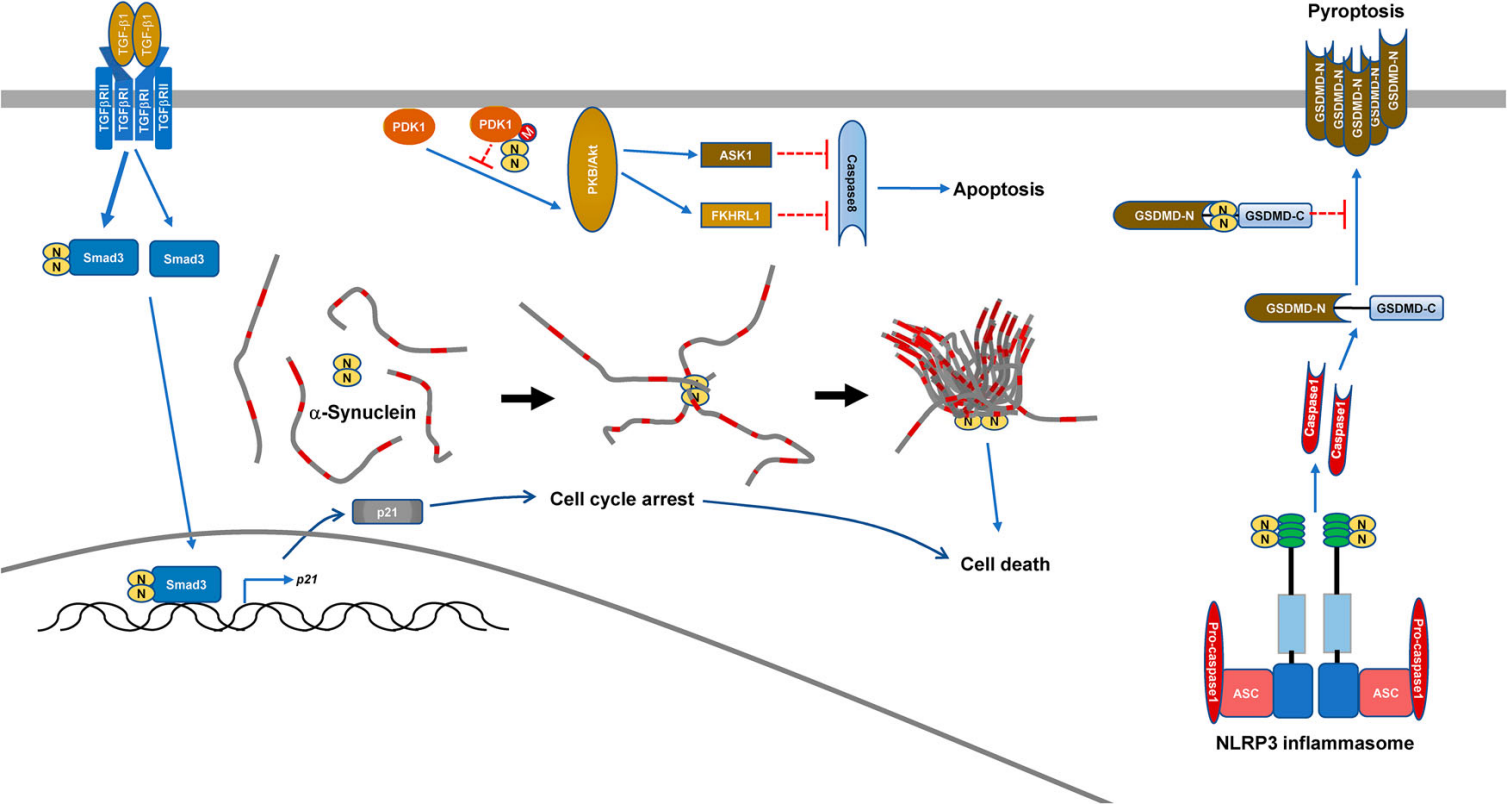
Modulation of cell death by SARS-CoV-2 N-protein.

In a word, SARS-CoV-2 N-protein can modulate different types of cell death in different manners (Table 1) (Figure 3), which also contributes to COVID-19 pathogenesis.

## Modulation of antiviral innate immunity by SARS-CoV-2 N-protein

In response to SARS-CoV-2 infection, retinoic acid-inducible gene I (RIG-I) and melanoma differentiation-associated protein (MDA5) sense viral RNA and subsequently translocate to the mitochondrion. Then they bind to the adaptor protein mitochondrial antiviral signaling (MAVS) to form a signalosome that leads to the phosphorylation and nuclear translocation of interferon regulatory factor (IRF) 3/7, which promotes the transcription of type I and III interferon genes. In addition, stress granules are formed in response to viral infection, causing G3BP1 to interact with RIG-I to promote its activation [44]. SARS-CoV-2 N-protein interacts with multiple proteins involved in the immune response and stress granule formation [11], which affects the production of antiviral interferons [29–31,39,45–48]. Several studies have showed that SARS-CoV-2 N-protein inhibits RNA virus-induced type I IFN production [29,31,39,40,46–48], even more potently than SARS-CoV N-protein [40], However, controversy reports on the effects of SARS-CoV-2 N-protein exist [30,45]. The paradox is possibly due to differences in cell lines used, N-protein sequences, and N-protein doses [29–31,39,40,45–48].

SARS-CoV-2 N-protein binds to RIG-I through the DExD/H domain of RIG-I, which hinders the recognition of viral RNA [48]. Furthermore, the interaction between SARS-CoV-2 N-protein and RIG-I can block the recruitment of tripartite motif protein 25, an E3 ligase that mediates K63-ubiquitination and subsequent activation of RIG-I, in a mode similar to N-proteins from MERS-CoV and SARS-CoV [29–31]. In addition, N-proteins of SARS-CoV and SARS-CoV-2, but not MERS-CoV, target G3BP1 to block stress granule formation [32,39], which prevents cofactors from enhancing RIG-I activation after SARS-CoV-2 infection [39]. The modulation of stress granules by SARS-CoV-2 N-protein have additional effects on RIG-I signaling. Even though one study observed no interaction between SARS-CoV-2 N-protein and MAVS [31], another study by a different group has detected that SARS-CoV-2 N-protein binds to MAVS in a LLPS-dependent manner, which inhibits the binding of MAVS to RIG-I and tripartite motif protein 31, an E3 ligase that mediates K63-ubiquitination and subsequent activation of MAVS [40].

After type I interferon production, type I interferons bind to their receptors and activate intracellular tyrosine kinases, resulting in subsequent phosphorylation and activation of signal transducer and activator of transcription (STAT) 1/2. The SARS-CoV-2 N-protein hinders type I interferon-induced STAT1/2 phosphorylation and nuclear translocation. Mechanistically, SARS-CoV-2 N-protein binds to STAT1/2 and blocks the interaction with upstream tyrosine kinases [30,41]. Intriguingly, SARS-CoV N-protein does not have such a role [34].

In mammals, the antiviral innate immunity may be directed by proteins or small RNAs [33]. After virus goes through infection and replication, the virus-derived double-stranded RNAs are recognized and cleaved by Dicer. Following cleavage by Dicer, the resulting small RNAs combine with the RNA-induced silencing complex. Endonucleases argonaute clade proteins are core components of the RNA-induced silencing complex, which uses the diced RNA as a template to bind and cleave viral RNA [33]. N-proteins from coronaviruses such as SARS-CoV and MERS-CoV are identified as viral suppressors of this mechanism [33]. Similarly, SARS-CoV-2 N-protein interacts with virus-derived double-stranded RNAs and then sequestrates them to suppress this pathway [42,43], thereby acting as a critical immune evasion factor of SARS-CoV-2.

Finally, human humoral fluid-phase pattern recognition molecule long pentraxin 3 binds to SARS-CoV-2 N-protein but shows no antiviral activity. Long pentraxin 3 is abundantly expressed by blood and lung myeloid cells. The serum concentration of long pentraxin 3 is associated with mortality of COVID-19 patients. The role of long pentraxin 3 in SARS-CoV-2 N-protein-mediated complement activation and cytokine production remain to be determined [49].

So SARS-CoV-2 N-protein can modulate antiviral innate immunity through multiple mechanisms (Table 1), which contributes to COVID-19 pathogenesis.

## SARS-CoV-2 N-protein activates the adaptive immunity

SARS-CoV-2 N-protein-specific CD4^+^ T cells are detected in most non-hospitalized and previously hospitalized COVID-19 subjects [50,51]. These CD4^+^ T cells express CD45RO, a marker of antigen experience [50]. A synchronous multipolar and antigen-specific T helper (Th) response can be detected immediately after a mild COVID-19 infection [51]. This response involves IL-2-production by T cells and correlates with virus-neutralizing antibody titers [52]. Also involved in the Th response is phenotypically stable Th1 or circulating follicular Th cells, which persist months [50,51]. In a portion of these subjects, the increased Th responses from memory circulating follicular Th cells correlated with sustained antibody production over time [50]. However, SARS-CoV-2 N-protein-specific Th1 cells are not always protective because even participants with higher frequencies of N-specific IFNγ^+^CD4^+^ T cells still required ICU care [53]. Furthermore, even long COVID patients with neurological symptoms showed enhanced activation of follicular Th cells as well as more Th1 cells, which is linked with increased levels of SARS-CoV-2 N-protein-specific antibodies [54].

Indeed, increased levels of SARS-CoV-2 N-protein-specific antibodies are correlated with disease severity [54–58]. Despite this, opposite trends have also been reported. In patients with cancer, the level of SARS-CoV-2 N-protein-specific antibodies is not correlated with peak viral load during acute COVID-19. Rather, a lower level of SARS-CoV-2 N-protein-specific antibodies is correlated with prolonged viral load [59]. Accordingly, a higher level of antibody against a specific epitope in SARS-CoV-2 N-protein is associated with moderate cases [58]. Thus, epitope-specific antibody responses to SARS-CoV-2 N-protein differentiate COVID-19 outcomes. Recently, it has been reported that extracellular SARS-CoV-2 N-protein released by SARS-CoV-2-infected cells binds to heparan sulfate and heparin on neighbouring cells by electrostatic high-affinity interaction. SARS-CoV-2 N-protein binds with high affinity to eleven human chemokines and thereby may sequester these chemokines. SARS-CoV-2 N-protein-specific antibodies bind to cell surface N-protein and thereby activate Fc receptor-expressing cells. These effects might either augment or reduce the severity of COVID-19 [60]. Nevertheless, the level of SARS-CoV-2 N-protein-specific antibodies rapidly declines in convalescence over time. By contrast, SARS-CoV-2 N-protein-specific IgM^+^ or IgG^+^ memory B cells continue to rise until 150 days [61]. B cell depletion is strongly correlated with prolonged SARS-CoV-2 viral load [59,62]. Thus, B cell memory is very important for COVID-19 control.

Multiple-functional CD8^+^ T cell responses against SARS-CoV-2 N-protein are associated with mild disease [63]. After recovery, severe COVID-19 subjects have SARS-CoV-2 N-protein-specific CD8^+^ with significantly higher cytotoxicity gene expression scores and increased inhibitory receptor expression, which results from higher antigen loads [64]. Post-acute symptoms are associated with a decline in SARS-CoV-2 N-protein-specific IFNγ^+^CD8^+^ T cells [63,64]. CD8^+^ T cells are essential for acute COVID-19 control and are especially important in patients lacking B cell function [64]. CD8^+^ T cells recognize small peptide epitopes (typically 8–10 amino acids) presented by human leukocyte antigen (HLA) molecules with varying affinity. Intriguingly, SARS-CoV-2 N-protein induces stronger CD8^+^ T cell responses in HLA-B07^+^ COVID-19-recovered individuals [65–67]. Epitope scanning indicates that a highly conserved epitope across circulating coronaviruses, N_105-113_, drives the immunodominant CD8^+^ T cell response in HLA-B07^+^ COVID-19-recovered individuals [65–67]. The corresponding CD8^+^ T cells have a highly diverse TCR repertoire, which is linked to the expansion of naïve precursors. Furthermore, the corresponding CD8^+^ T cells can be maintained for months with preserved antiviral efficacy to various SARS-CoV-2 strains [65–67].

## SARS-CoV-2 N-protein as a target for vaccine development

Most authorized SARS-CoV-2 vaccines are based on the S-protein of the ancestral SARS-CoV-2, which elicits neutralizing antibody responses. However, Omicron contains many mutations located in the S-protein, especially in the receptor-binding domain, which is the major target of neutralizing antibody responses. It is not surprising that Omicron escapes the immune response to these vaccines. Vaccination of BALB/c mice by different routes using SARS-CoV-2 N-protein elicited robust antibody and IFNγ production [56,68]. The SARS-CoV-2 N-protein has become a target for vaccine development since the majority of the putative T cell specific epitopes are conserved in the variants of concern (Table 2) [69–81].

**Table 2.** SARS-CoV-2-N-based vaccines under development.

	Vaccine name	N-protein usage	Adverse effects in clinical studies	Protective effects in preclinical studies	Antibody responses	T cell responses	References
1	COH04S1 (a synthetic multiantigen MVA-based vaccine)	Full length N-protein from SARS-CoV-2 ancestral virus, with co-expression of S protein.	Grade 3 fever was observed in one participant who received intramuscular injection of low-dose COH04S1 and placebo, grade 2 anxiety or fatigue was seen in one participant who received medium-dose COH04S1, no severe adverse events were reported in an open-label and randomized, phase 1 trial.	Intramuscular and intranasal vaccination protected Syrian hamsters from weight loss, low respiratory tract infection, and lung pathology following challenge with SARS-CoV-2 ancestral virus and Beta and Delta variants. 2 doses or 1 dose intramuscular vaccination protected African green monkey NHP from lower and upper respiratory tract infection following challenge with SARS-CoV-2 ancestral virus.	Both preclinical and clinical studies: robust binding antibodies to N-protein, robust but rapidly declined virus-neutralizing antibodies which showed reduced cross-reactivity.	Preclinical studies: robust mucosal and systemic IFNγ- and IL-2-expressing N-protein-specific T cells with no or marginally detectable IL-4-expressing N-protein-specific T cells. Clinical studies: blood N-specific T cells elicited in COH04S1-vaccinees demonstrated potent and equivalent cross-reactivity against ancestral SARS-CoV-2 and Delta and Omicron variants.	[69–72]
2	N/A (a vaccine with the LVS Δ*capB* vector, a highly attenuated replicating intracellular bacterium)	Full length N-protein from SARS-CoV-2 ancestral virus, with co-expression of M protein.	N/A	Intradermal and intranasal vaccination protected Syrian hamsters from weight loss, low respiratory tract infection, and lung pathology following challenge with SARS-CoV-2 ancestral virus.	Preclinical study: protection correlated with binding antibodies to N-protein.	N/A	[73]
3	N/A (a replication-deficient human adenovirus type 5 vector vaccine)	Full length N-protein from SARS-CoV-2 ancestral virus.	N/A	Intravenous vaccination of Syrian hamsters and K18-hACE2 transgenic C57 BL/6 mice conferred protection, as defined by reduced weight loss and viral load, following challenge with SARS-CoV-2 ancestral virus.	N/A	Preclinical study: rapid and persistent N-specific CD8^+^ T cell responses in the respiratory mucosa.	[74]
4	GX-19N (a recombinant DNA vaccine)	Full length N-protein from SARS-CoV-2 ancestral virus, with co-expression of S protein.	The vaccines were delivered intramuscularly using an electroporator. All solicited adverse events were mild; no serious vaccine-related adverse events were detected.	N/A	Clinical study: no information about binding antibodies to N-protein, virus-neutralizing antibodies were weaker than those induced by commercial vaccines.	Clinical study: broad N-protein-specific T-cell responses in the blood.	[75]
5	SpiN (a fusion protein vaccine)	Full length N-protein from SARS-CoV-2 ancestral virus, in fusion with RBD of S protein.	N/A	Intramuscularimmunization of Syrian hamsters and K18-ACE2 transgenic C57 BL/6 mice with poly I:C as the adjuvant promoted robust resistance to SARS-CoV-2 ancestral virus, as indicated by viral load, lung inflammation, weight loss, and reduction of lethality. This vaccine also protected K18-ACE2 transgenic C57 BL/6 mice against infection with Delta and Omicron variants.	Preclinical study: robust binding antibodies to N-protein, no virus-neutralizing antibodies.	Preclinical study: robust mucosal and systemic IFNγ response by N-protein-specific T cells.	[76]
6	Tri:HuAd (a replication-deficient human adenovirus type 5 vector vaccine) or Tri:ChAd (a replication-deficient chimpanzee adenovirus type 68 vector vaccine)	Full length N-protein from SARS-CoV-2 ancestral virus, in fusion witha highly conserved region of RNA-dependent RNA polymerase and with co-expression of S1 domain of S protein.	N/A	Intranasal, but not intramuscular, immunization of BALB/c mice provided potent B- and T-cell-dependent protection from the lethal dose infection with mouse-adapted strain of SARS-CoV-2 ancestral virus. Single-dose intranasal immunization with ChAd-vectored trivalent COVID-19 vaccine protected against lethal infection by SARS-CoV-2 variants of concern.	Preclinical study: no information about binding antibodies to N-protein, robust virus-neutralizing antibodies.	Preclinical study: intranasal vaccination induced robust mucosal and systemic IFNγ-, TNFα-, or IL-2-expressing monofunctional N-protein-specific CD8^+^ T cells, robust mucosal and systemic IFNγ-expressing N-protein-specific CD4^+^ T cells, and respiratory mucosal tissue-resident memory T cell responses.	[77]
7	N/A (a replication-deficient human adenovirus type 5 vector vaccine)	Full length N-protein from SARS-CoV-2 ancestral virus, with co-expression of S protein.	N/A	Intramuscular vaccination of Syrian hamsters and K18-hACE2 transgenic C57 BL/6 mice conferred acute protection in both the lung and brain following challenge with SARS-CoV-2 ancestral virus.	Preclinical study: robust binding antibodies to N-protein, no information about virus-neutralizing antibodies.	Preclinical study: robust local (lung and brain) and systemic IFNγ-expressing N-protein-specific CD8^+^ T cells, robust systemic IFNγ-expressing N-protein-specific CD4^+^ T cells.	[78]
8	N/A (a recombinant protein vaccine with flagellin and cyclic GMP-AMP as adjuvants)	Full length N-protein from SARS-CoV-2 ancestral virus, with co-expression of prefusion-full S protein	N/A	Intranasal vaccination of K18-hACE2 transgenic C57 BL/6 mice protected against lethal SARS-CoV-2 ancestral virus challenge, with superior protection in the upper respiratory tract compared with that in mice immunized with an inactivated vaccine.	Preclinical study: robust binding antibodies to N-protein, robust IgA^+^ B cells, robust virus-neutralizing antibodies which reduced cross-reactivity.	Preclinical study: robust mucosal and systemic IFNγ- or IL-4-expressing N-protein-specific T cells with equivalent cross-reactivity against ancestral SARS-CoV-2 and Beta and Delta variants, more mucosal CD8^+^ T cells, respiratory mucosal tissue-resident CD4^+^ and CD8^+^ T cells.	[79]
9	UB-612 (a multitope subunit vaccine)	Rationally designed promiscuous peptides representing sarbecovirus conserved CD4^+^ ad CD8^+^ T cell epitopes on N, M, and S2 proteins, and S1-RBD-sFc protein.	No vaccine-related serious adverse events were recorded after intramuscular vaccination. The most common solicited adverse events were injection site pain and fatigue, mostly mild and transient.	N/A	Clinical studies: no information about N-protein-specific antibodies, potent neutralizing titers against ancestral SARS-CoV-2 virus, Delta, Omicron, and other variants of concern, but the virus-neutralizing antibodies were long-lasting as revealed with the live ancestral SARS-CoV-2 virus.	Clinical studies: no information about N-protein-specific T cells, restimulation of peripheral blood mononuclear cells with designer peptide antigens revealed long-lasting robust Th1-predominant cell response as measured by IFN-γ and IL-4 ELISpot.	[80]
10	N/A (a nucleoside-modified mRNA vaccine)	A mRNA vaccine expressing fulll length N-protein from SARS-CoV-2 ancestral virus, alone or in combination withthe clinically proven S-expressing mRNA vaccine.	N/A	Intramuscular, but not intranasal, immunization of BALB/c mice and Syrian hamsters with N-protein mRNA induced modest control of mouse-adapted SARS-CoV-2, as indicated by viral load and body weight. In BALB/c mice, dual S- and N-protein mRNA vaccination further lowered viral load. In Syrian hamsters, dual S- and N-protein mRNA vaccination not only induced more robust control of the Delta and Omicron variants in the lungs but also provided enhanced protection in the upper respiratory tract.	Preclinical study: intramuscular vaccination with N-protein mRNA alone induced robust binding antibodies to N-protein but no neutralizing activity, dual S- and N-protein mRNA vaccination augmented serum neutralizing antibody activities.	Preclinical study: intramuscular vaccination with N-protein mRNA alone induced robust systemic N-protein-specific CD4^+^ and CD8^+^ T cell responses for IFNγ-, TNFα-, and IL-2, dual S- and N-protein mRNA vaccination augmented systemic S-protein-specific CD8^+^ T cell response.	[81]

Note: N/A, non-available; NHP, non-human primates.

Up to now, 9 of the 10 SARS-CoV-2 N-based vaccines employ full length N-protein from SARS-CoV-2 ancestral virus, alone or in combination with other SARS-CoV-2 structural proteins [69–81]. Although with different compositions (vector-based [69,70,73,74,77,78], recombinant protein [76,79], or mRNA [81]) and different vaccination routes (intramuscular [69,70,76,78,81], intradermal [73], intranasal [73,77,79], or intravenous [74]), these 9 vaccines elicited protective effects in all the preclinical studies with SARS-CoV-2 ancestral virus infection. More importantly, vaccines with full length N-protein conferred protection against infection by the SARS-CoV-2 variants of concern [69,70,76,77,81]. Additionally, these vaccines with full length N-protein demonstrated high tolerability in clinical trials [71,72,75] and 6 of the 9 vaccines elicited production of antibodies with robust binding to the N-protein (Table 2) [69–79,81].

Even though one study reported that protection correlated with antibodies against the N-protein [73], it is unlikely that antibodies are essential for protection against infection by ongoing SARS-CoV-2 variants. There are at least 3 reasons for this notion: (1) Immunization with N-protein mRNA induced modest control of mouse-adapted SARS-CoV-2, as indicated by viral load and body weight in BALB/c mice and Syrian hamsters. In BALB/c mice, dual S- and N-protein mRNA vaccination can lower viral load. While in Syrian hamsters, dual S- and N-protein mRNA vaccination induced not only more robust control of the Delta and Omicron variants in lungs but also increased protection in the upper respiratory tract. Under these conditions, robust N-specific binding antibodies were detected without neutralizing activity [81]. (2) The virus-neutralizing antibodies elicited by multiantigen COH04S1-vaccines using modified Vaccinia Ankara (MVA) vector showed reduced cross-reactivity but vaccination with COH04S1 promoted robust resistance to Beta and Delta variants [70]; (3) SpiN, a protein vaccine with full length N-protein fused with the receptor binding domain (RBD) of the S-protein, protected K18-ACE2 transgenic C57 BL/6 mice against infection with Delta and Omicron variants, although without detectable neutralizing antibodies [76].

Overall, the T cell responses elicited by vaccines with full length N-protein should be pivotal for protection against infection by SARS-CoV-2 variants of concern [69–79,81]. The available data demonstrate robust local and systemic IFNγ responses by N-protein-specific T cells and also responses from respiratory mucosal tissue-resident memory T cells. Because T cell epitopes in SARS-CoV-2 N-protein are highly conserved, these N-protein-specific T cells show equivalent cross-reactivity against ancestral SARS-CoV-2 and variants of concern (Table 2) [69–79]. Thus, these N-protein-specific T cells may constitute a critical second line of defense for providing long-term protection against SARS-CoV-2 variants. Indeed, in *vivo* CD8^+^ T cell depletion in Syrian hamsters after vaccination with N-protein mRNA identifies a role for CD8^+^ T cells in viral control as well as protection against body weight loss following Omicron challenge [81].

From this point of view, the usage of T cell-specific epitopes in the SARS-CoV-2 N-protein may be a better choice than full length protein for vaccine development. This strategy may also avoid the possible adverse effects of binding antibodies to N-protein [54–58]. Indeed, one of the 10 vaccines with N-protein usage employed the strategy of rationally designed T cell epitopes [80]. Although information about N-protein-specific T cells is unavailable, long-lasting T cell immunity against Delta and Omicron variants has been observed [80]. This warrants a large-scale field trial for evaluation.

## Conclusions

The SARS-CoV-2 N-protein plays pivotal roles in inflammation, cell death, innate antiviral immunity, and adaptive antiviral immunity. Further research is required to clarify how the SARS-CoV-2 N-protein modulates inflammation, cell death, and innate antiviral immunity. Of importance is to identify the key motifs involved. Additionally, even though SARS-CoV-2 N-based vaccines showed protective effects, it should be noted that SARS-CoV-2 N-protein-specific T cells may cause inflammatory related injury, especially after repeated boosting. Thus, it is essential to identify protective T cell-specific epitopes in the SARS-CoV-2 N-protein according to different HLA genotypes.

## References

[1] World Health Organization. Rolling updates on coronavirus disease (COVID-19). Available at: https://www.who.int/emergencies/diseases/novel-coronavirus-2019

[2] Huang C, Wang Y, Li X, et al. Clinical features of patients infected with 2019 novel coronavirus in Wuhan, China. Lancet. 2020;395:497–506. 31986264 10.1016/S0140-6736(20)30183-5 PMC7159299

[3] Hoffmann M, Kleine-Weber H, Schroeder S, et al. SARS-CoV-2 cell entry depends on ACE2 and TMPRSS2 and is blocked by a clinically proven protease inhibitor. Cell. 2020;181:271–280.e8. 32142651 10.1016/j.cell.2020.02.052 PMC7102627

[4] Finkel Y, Mizrahi O, Nachshon A, et al. The coding capacity of SARS-CoV-2. Nature. 2021;589:125–130. 32906143 10.1038/s41586-020-2739-1

[5] Kang S, Yang M, Hong Z, et al. Crystal structure of SARS-CoV-2 nucleocapsid protein RNA binding domain reveals potential unique drug targeting sites. Acta Pharm Sin B. 2020;10:1228–1238. 32363136 10.1016/j.apsb.2020.04.009 PMC7194921

[6] Peng Y, Du N, Lei Y, et al. Structures of the SARS-CoV-2 nucleocapsid and their perspectives for drug design. EMBO J. 2020;39:e105938. 32914439 10.15252/embj.2020105938 PMC7560215

[7] Jia Z, Liu C, Chen Y, et al. Crystal structure of the SARS-CoV-2 nucleocapsid protein C-terminal domain and development of nucleocapsid-targeting nanobodies. FEBS J. 2022;289:3813–3825. 34665939 10.1111/febs.16239 PMC8646419

[8] Iserman C, Roden CA, Boerneke MA, et al. Genomic RNA elements drive phase separation of the SARS-CoV-2 nucleocapsid. Mol Cell. 2020;80:1078–1091.e6. 33290746 10.1016/j.molcel.2020.11.041 PMC7691212

[9] Perdikari TM, Murthy AC, Ryan VH, et al. SARS-CoV-2 nucleocapsid protein phase-separates with RNA and with human hnRNPs. EMBO J. 2020;39:e106478. 33200826 10.15252/embj.2020106478 PMC7737613

[10] Biswal M, Lu J, Song J. SARS-CoV-2 nucleocapsid protein targets a conserved surface groove of the NTF2-like domain of G3BP1. J Mol Biol. 2022;434:167516. 35240128 10.1016/j.jmb.2022.167516 PMC8882607

[11] Somasekharan SP, Gleave M. SARS-CoV-2 nucleocapsid protein interacts with immunoregulators and stress granules and phase separates to form liquid droplets. FEBS Lett. 2021;595:2872–2896. 34780058 10.1002/1873-3468.14229 PMC8652540

[12] Li Y, Lu S, Gu J, et al. SARS-CoV-2 impairs the disassembly of stress granules and promotes ALS-associated amyloid aggregation. Protein Cell. 2022;13:602–614. 35384603 10.1007/s13238-022-00905-7 PMC8983322

[13] Toldo S, Bussani R, Nuzzi V, et al. Inflammasome formation in the lungs of patients with fatal COVID-19. Inflamm Res. 2021;70:7–10. 33079210 10.1007/s00011-020-01413-2 PMC7572246

[14] Rodrigues TS, de Sa KSG, Ishimoto AY, et al. Inflammasomes are activated in response to SARS-CoV-2 infection and are associated with COVID-19 severity in patients. J Exp Med. 2021;218:e20201707. 33231615 10.1084/jem.20201707 PMC7684031

[15] Pan P, Shen M, Yu Z, et al. SARS-CoV-2 N protein promotes NLRP3 inflammasome activation to induce hyperinflammation. Nat Commun. 2021;12:4664. 34341353 10.1038/s41467-021-25015-6 PMC8329225

[16] Wu Y, Ma L, Cai S, et al. RNA-induced liquid phase separation of SARS-CoV-2 nucleocapsid protein facilitates NF-κB hyper-activation and inflammation. Signal Transduct Target Ther. 2021;6:167. 33895773 10.1038/s41392-021-00575-7 PMC8065320

[17] Chen L, Guan W, Qiu ZE, et al. SARS-CoV-2 nucleocapsid protein triggers hyperinflammation via protein-protein interaction-mediated intracellular Cl^−^ accumulation in respiratory epithelium. Signal Transduct Target Ther. 2022;7:255. 35896532 10.1038/s41392-022-01048-1 PMC9328007

[18] Zhao X, Nicholls JM, Chen YG. Severe acute respiratory syndrome-associated coronavirus nucleocapsid protein interacts with Samd3 and modulates transforming growth factor-beta signaling. J Biol Chem. 2008;283:3272–3280. 18055455 10.1074/jbc.M708033200 PMC8740907

[19] Olea B, Albert E, Torres I, et al. SARS-CoV-2 N-antigenemia in critically ill adult COVID-19 patients: frequency and association with inflammatory and tissue-damage biomarkers. J Med Virol. 2022;94:222–228. 34449894 10.1002/jmv.27300 PMC8662104

[20] Yokoyama R, Kurano M, Nakano Y, et al. Association of the serum levels of the nucleocapsid antigen of SARS-CoV-2 with the diagnosis, disease severity, and antibody titers in patients with COVID-19: a retrospective cross-sectional study. Front Microbiol. 2021;12:791489. 34956158 10.3389/fmicb.2021.791489 PMC8696188

[21] Favresse J, Bayart JL, David C, et al. Nucleocapsid serum antigen determination in SARS-CoV-2 infected patients using the single molecule array technology and prediction of disease severity. J Infect. 2022;84:e4–e6. 35074508 10.1016/j.jinf.2022.01.023 PMC8779852

[22] Gao T, Zhu L, Liu H, et al. Highly pathogenic coronavirus N protein aggravates inflammation by MASP-2-mediated lectin complement pathway overactivation. Signal Transduct Target Ther. 2022;7:318. 36100602 10.1038/s41392-022-01133-5 PMC9470675

[23] Ali YM, Ferrari M, Lynch NJ, et al. Lectin pathway mediates complement activation by SARS-CoV-2 proteins. Front Immunol. 2021;12:714511. 34290717 10.3389/fimmu.2021.714511 PMC8287855

[24] Qian Y, Lei T, Patel PS, et al. Direct activation of endothelial cells by SARS-CoV-2 nucleocapsid protein is blocked by simvastatin. J Virol. 2021;95:e0139621. 34549987 10.1128/JVI.01396-21 PMC8577385

[25] Xia J, Tang W, Wang J, et al. SARS-CoV-2 N protein induces acute lung injury in mice via NF-κB activation. Front Immunol. 2021;12:791753. 34950152 10.3389/fimmu.2021.791753 PMC8688532

[26] Nakayama EE, Kubota-Koketsu R, Sasaki T, et al. Anti-nucleocapsid antibodies enhance the production of IL-6 induced by SARS-CoV-2 N protein. Sci Rep. 2022;12:8108. 35577892 10.1038/s41598-022-12252-y PMC9109953

[27] Freda CT, Yin W, Ghebrehiwet B, et al. SARS-CoV-2 proteins regulate inflammatory, thrombotic and diabetic responses in human arterial fibroblasts. Clin Immunol. 2021;227:108733. 33895357 10.1016/j.clim.2021.108733 PMC8061629

[28] Kao MS, Yang JH, Balasubramaniam A, et al. Colonization in nasal cavities by *staphylococcus epidermidis* mitigates SARS-CoV-2 nucleocapsid phosphoprotein-induced interleukin (IL)-6 in the lung. Microb Biotechnol. 2022;15:1984–1994. 35426250 10.1111/1751-7915.13994 PMC9111282

[29] Savellini GG, Anichini G, Gandolfo C, et al. SARS-CoV-2 N protein targets TRIM25-mediated RIG-I activation to suppress innate immunity. Viruses. 2021;13:1439. 34452305 10.3390/v13081439 PMC8402637

[30] Zhao Y, Sui L, Wu P, et al. A dual-role of SARS-CoV-2 nucleocapsid protein in regulating innate immune response. Signal Transduct Target Ther. 2021;6:331. 34471099 10.1038/s41392-021-00742-w PMC8409078

[31] Oh SJ, Shin OS. SARS-CoV-2 nucleocapsid protein targets RIG-I-like receptor pathways to inhibit the induction of interferon response. Cells. 2021;10:530. 33801464 10.3390/cells10030530 PMC7999926

[32] Zheng ZQ, Wang SY, Xu ZS, et al. SARS-CoV-2 nucleocapsid protein impairs stress granule formation to promote viral replication. Cell Discov. 2021;7:38. 34035218 10.1038/s41421-021-00275-0 PMC8147577

[33] Cui L, Wang H, Ji Y, et al. The nucleocapsid protein of coronaviruses acts as a viral suppressor of RNA silencing in mammalian cells. J Virol. 2015;89:9029–9043. 26085159 10.1128/JVI.01331-15 PMC4524063

[34] Kopecky-Bromberg SA, Martinez-Sobrido L, Frieman M, et al. Severe acute respiratory syndrome coronavirus open Reading frame (ORF) 3b, ORF6, and nucleocapsid proteins functions as interferon antagonists. J Virol. 2007;81:548–557. 17108024 10.1128/JVI.01782-06 PMC1797484

[35] Wang W, Chen J, Hu D, et al. SARS-CoV-2 N protein induces acute kidney injury via Smad3-dependent G1 cell cycle arrest mechanism. Adv Sci. 2022;9:2103248. 10.1002/advs.202103248 PMC8787402 34813685

[36] Ma J, Zhu F, Zhao M, et al. SARS-CoV-2 nucleocapsid suppresses host pyroptosis by blocking Gasdermin D cleavage. EMBO J. 2021;40:e108249. 34296442 10.15252/embj.2021108249 PMC8420271

[37] Ren Y, Wang A, Fang Y, et al. SARS-CoV-2 membrane glycoprotein M triggers apoptosis with the assistance of nucleocapsid protein N in cells. Front Cell Infect Microbiol. 2021;11:706252. 34513728 10.3389/fcimb.2021.706252 PMC8425412

[38] Semerdzhiev SA, Fakhree MAA, Segers-Nolten I, et al. Interaction between SARS-CoV-2 N-protein and α-synuclein accelerate amyloid formation. ACS Chem Neurosci. 2022;13:143–150. 34860005 10.1021/acschemneuro.1c00666 PMC8739828

[39] Zheng Y, Deng J, Han L, et al. SARS-CoV-2 NSP5 and N protein counteract the RIG-I signaling pathway by suppressing the formation of stress granules. Signal Transduct Target Ther. 2022;7:22. 35075101 10.1038/s41392-022-00878-3 PMC8785035

[40] Wang S, Dai T, Qin Z, et al. Targeting liquid-liquid phase separation of SARS-CoV-2 nucleocapsid protein promotes innate antiviral immunity by elevating MAVS activity. Nat Cell Biol. 2021;23:718–732. 34239064 10.1038/s41556-021-00710-0

[41] Mu J, Fang Y, Yang Q, et al. SARS-CoV-2 N protein antagonizes type I interferon signaling by suppressing phosphorylation and nuclear translocation of STAT1 and STAT2. Cell Discov. 2020;6:65. 32953130 10.1038/s41421-020-00208-3 PMC7490572

[42] Mu J, Xu J, Zhang L, et al. SARS-CoV-2-encoded nucleocapsid protein acts as a viral suppressor of RNA interference in cells. Sci China Life Sci. 2020;63:1413–1416. 32291557 10.1007/s11427-020-1692-1 PMC7154568

[43] Chen YM, Wei JL, Qin RS, et al. Folic acid: a potential inhibitor against SARS-CoV-2 nucleocapsid protein. Pharm Biol. 2022;60:862–878. 35594385 10.1080/13880209.2022.2063341 PMC9132477

[44] Kim SS, Sze L, Liu C, et al. The stress granule protein G3BP1 binds viral dsRNA and RIG-I to enhance interferon-beta response. J Biol Chem. 2019;294:6430–6438. 30804210 10.1074/jbc.RA118.005868 PMC6484135

[45] Xia H, Cao Z, Xie X, et al. Evasion of type I interferon by SARS-CoV-2. Cell Rep. 2020;33:108234. 32979938 10.1016/j.celrep.2020.108234 PMC7501843

[46] Lei X, Dong X, Ma R, et al. Activation and evasion of type I interferon responses by SARS-CoV-2. Nat Commun. 2020;11:3810. 32733001 10.1038/s41467-020-17665-9 PMC7392898

[47] Li JY, Liao CH, Wang Q, et al. The ORF6, ORF8 and nucleocapsid proteins of SARS-CoV-2 inhibit type I interferon signaling pathway. Virus Res. 2020;286:198074. 32589897 10.1016/j.virusres.2020.198074 PMC7309931

[48] Chen K, Xiao F, Hu D, et al. SARS-CoV-2 nucleocapsid protein interacts with RIG-I and represses RIG-mediated IFN-β production. Viruses. 2021;13:47. 10.3390/v13010047 PMC7823417 33396605

[49] Stravalaci M, Pagani I, Paraboschi EM, et al. Recognition and inhibition of SARS-CoV-2 by humoral innate immunity pattern recognition molecules. Nat Immunol. 2022;23:275–286. 35102342 10.1038/s41590-021-01114-w

[50] Nelson RW, Chen Y, Venezia OL, et al. SARS-CoV-2 epitope-specific CD4^+^ memory T cell responses across COVID-19 disease severity and antibody durability. Sci Immunol. 2022;7:eabl9464. 35857584 10.1126/sciimmunol.abl9464 PMC9097883

[51] Martner A, Wiktorin HG, Törnell A, et al. Transient and durable T cell reactivity after COVID-19. Proc Natl Acad Sci U S A. 2022;119:e2203659119. 35858456 10.1073/pnas.2203659119 PMC9335198

[52] Koerber N, Priller A, Yazici S, et al. Dynamics of spike-and nucleocapsid specific immunity during long-term follow-up and vaccination of SARS-CoV-2 convalescents. Nat Commun. 2022;13:153. 35013191 10.1038/s41467-021-27649-y PMC8748966

[53] Peluso MJ, Deitchman AN, Torres L, et al. Long-term SARS-CoV-2-specific immune and inflammatory responses in individuals recovering from COVID-19 with and without post-acute symptoms. Cell Rep. 2021;36:109518. 34358460 10.1016/j.celrep.2021.109518 PMC8342976

[54] Visvabharathy L, Hanson B, Orban Z, et al. Neuro-COVID long-haulers exhibit broad dysfunction in T cell memory generation and response to vaccination. MedRxiv*.* 2021;2021.08.08.21261763.

[55] Yang L, Xu Q, Yang B, et al. Igg antibody titers against SARS-CoV-2 nucleocapsid protein correlate with the severity of COVID-19 patients. BMC Microbiol. 2021;21:351. 34922455 10.1186/s12866-021-02401-0 PMC8683808

[56] Feng W, Xiang Y, Wu L, et al. Nucleocapsid protein of SARS-CoV-2 is a potential target for developing new generation of vaccine. J Clin Lab Anal. 2022;36:e24479. 35527696 10.1002/jcla.24479 PMC9169192

[57] Sen AR, Sanders EC, Gabriel KN, et al. Predicting COVID-19 severity with a specific nucleocapsid antibody plus disease risk factor score. mSphere. 2021;6:e00203–21. 33910993 10.1128/mSphere.00203-21 PMC8092137

[58] Voss C, Esmail S, Liu X, et al. Epitope-specific antibody responses differentiate COVID-19 outcomes and variants of concern. JCI Insight. 2021;6:e148855. 34081630 10.1172/jci.insight.148855 PMC8410046

[59] Lyudovyk O, Kim JY, Qualls D, et al. Impaired humoral immunity is associated with prolonged COVID-19 despite robust CD8 T cell responses. Cancer Cell. 2022;40:738–753.e5. 35679859 10.1016/j.ccell.2022.05.013 PMC9149241

[60] López-Muñoz AD, Kosik I, Holly J, et al. Cell surface SARS-CoV-2 nucleocapsid protein modulates innate and adaptive immunity. Sci Adv. 2022;8:eabp9770. 35921414 10.1126/sciadv.abp9770 PMC9348789

[61] Hartley GE, Edwars ESJ, Aui PM, et al. Rapid generation of durable B cell memory to SARS-CoV-2 spike and nucleocapsid proteins in COVID-19 and convalescence. Sci Immunol. 2020;5:eabf8891. 33443036 10.1126/sciimmunol.abf8891 PMC7877496

[62] Lee CY, Shah MK, Hoyos D, et al. Prolonged SARS-CoV-2 infection in patients with lymphoid malignancies. Cancer Discov. 2022;12:62–73. 34753749 10.1158/2159-8290.CD-21-1033 PMC8758535

[63] Peng Y, Mentzer AJ, Liu G, et al. Broad and strong memory CD4^+^ and CD8^+^ T cells induced by SARS-CoV-2 in UK convalescent individuals following COVID-19. Nat Immunol. 2020;21:1336–1345. 32887977 10.1038/s41590-020-0782-6 PMC7611020

[64] Bange EM, Han NA, Wileyto P, et al. CD8(+) t cells contribute to survival in patients with COVID-19 and hematologic cancer. Nat Med. 2021;27:1280–1289. 34017137 10.1038/s41591-021-01386-7 PMC8291091

[65] Peng Y, Felce SL, Dong D, et al. An immunodominant NP_105-113_-B*07:02 cytotoxic T cell response controls viral replication and is associated with less severe COVID-19 disease. Nat Immunol. 2022;23:50–61. 34853448 10.1038/s41590-021-01084-z PMC8709787

[66] Lineburg KE, Grant EJ, Swaminathan S, et al. CD8^+^ t cells specific for an immunodominant SARS-CoV-2 nucleocapsid epitope cross-react with selective seasonal coronaviruses. Immunity. 2021;54:1055–1065.e5. 33945786 10.1016/j.immuni.2021.04.006 PMC8043652

[67] Nguyen THO, Rowntree LC, Petersen J, et al. CD8^+^ t cells specific for an immunodominant SARS-CoV-2 nucleocapsid epitope display high naïve precursors frequency and TCR promiscuity. Immunity. 2021;54:1066–1082.e5. 33951417 10.1016/j.immuni.2021.04.009 PMC8049468

[68] He J, Huang J, Zhang Y, et al. SARS-CoV-2 nucleocapsid protein intranasal inoculation induces local and systemic T cell responses in mice. J Med Virol. 2021;93:1923–1925. 33386773 10.1002/jmv.26769

[69] Chiuppesi F, Nguyen VH, Park Y, et al. Synthetic multiantigen MVA vaccine COH04S1 protects against SARS-CoV-2 in Syrian hamsters and non-human primates. NPJ Vaccines. 2022;7:7. 35064109 10.1038/s41541-022-00436-6 PMC8782996

[70] Wussow F, Kha M, Faircloth K, et al. COH04S1 and beta sequence-modified vaccine protect hamsters from SARS-CoV-2 variants. iScience. 2022;25:104457. 35634578 10.1016/j.isci.2022.104457 PMC9126022

[71] Chiuppesi F, Zaia JA, Frankel PH, et al. Safety and immunogenicity of a synthetic multiantigen modified vaccinia virus Ankara-based COVID-19 vaccine (COH04S1): an open-label and randomised, phase 1 trial. Lancet Microbe. 2022;3:e252–e264. 35287430 10.1016/S2666-5247(22)00027-1 PMC8906816

[72] Chiuppesi F, Zaia JA, Frankel PH, et al. Vaccine-induced spike- and nucleocapsid-specific cellular responses maintain potent cross-reactivity to SARS-CoV-2 Delta and Omicron variants. iScience. 2022;25:104745. 35846380 10.1016/j.isci.2022.104745 PMC9272674

[73] Jia Q, Bielefeldt-Ohmann H, Maison RM, et al. Replicating bacterium-vectored vaccine expressing SARS-CoV-2 membrane and nucleocapsid proteins protects against severe COVID-19-like disease in hamsters. NPJ Vaccines. 2021;6:47. 33785745 10.1038/s41541-021-00321-8 PMC8009914

[74] Matchett WE, Joag V, Stolley JM, et al. Nucleocapsid vaccine elicits spike-independent SARS-CoV-2 protective immunity. J Immunol. 2021;207:376–379. 34193597 10.4049/jimmunol.2100421 PMC8516699

[75] Ahn JY, Lee J, Suh YS, et al. Safety and immunogenicity of two recombinant DNA COVID-19 vaccines containing the coding regions of the spike or spike and nucleocapsid proteins: an interim analysis of two open-label, non-randomised, phase 1 trials in healthy adults. Lancet Microbe. 2022;3:e173–e183. 35156068 10.1016/S2666-5247(21)00358-X PMC8824525

[76] Castro JT, Azevedo P, Fumagalli MJ, et al. Promotion of neutralizing antibody-independent immunity to wild-type and SARS-CoV-2 variants of concern using an RBD-nucleocapsid fusion protein. Nat Commun. 2022;13:4831. 35977933 10.1038/s41467-022-32547-y PMC9382605

[77] Afkhami S, D’Agostino MR, Zhang A, et al. Respiratory mucosal delivery of next-generation COVID-19 vaccine provides robust protection against both ancestral and variant strains of SARS-CoV-2. Cell. 2022;185:896–915.e19. 35180381 10.1016/j.cell.2022.02.005 PMC8825346

[78] Dangi T, Class J, Palacio N, et al. Combining spike- and nucleocapsid-based vaccines improves distal control of SARS-CoV-2. Cell Rep. 2021;36:109664. 34450033 10.1016/j.celrep.2021.109664 PMC8367759

[79] Jiang W, Shi L, Cai L, et al. A two-adjuvant multiantigen candidate vaccine induces superior protective immune responses against SARS-CoV-2 challenge. Cell Rep. 2021;37:110112. 34863353 10.1016/j.celrep.2021.110112 PMC8610932

[80] Wang CY, Hwang KP, Kuo HK, et al. A multitope SARS-CoV-2 vaccine provides long-lasting B cell and T cell immunity against Delta and Omicron variants. J Clin Invest. 2022;132:e157707. 35316221 10.1172/JCI157707 PMC9106357

[81] Hajnik RL, Plante JA, Liang Y, et al. Dual spike and nucleocapsid mRNA vaccination confer protection against SARS-CoV-2 Omicron and Delta variants in preclinical models. Sci Transl Med. 2022;14:eabq1945. 36103514 10.1126/scitranslmed.abq1945 PMC9926941

